# Quintic refractive index profile-based funnel-shaped silicon antireflective structures for enhanced photodetector performance

**DOI:** 10.1038/s41598-024-61156-6

**Published:** 2024-05-06

**Authors:** Beom-Jun Kim, Min-Seung Jo, Jae-Soon Yang, Myung-Kun Chung, Sung-Ho Kim, Jun-Bo Yoon

**Affiliations:** 1 School of Electrical Engineering, Korea Advanced Institute of Science and Technology (KAIST), 291 Daehak-ro, Yuseong-gu, Daejeon, 34141 Republic of Korea; 2 Center for Bio-Integrated Electronics, Northwestern University, 633 Clark St, Evanston, IL 60208 USA

**Keywords:** Optical materials and structures, Electrical and electronic engineering

## Abstract

Antireflection, vital in optoelectronics devices such as solar cells and photodetectors, reduces light reflection and increases absorption. Antireflective structures (ARS), a primary method by which to realize this effect, control the refractive index (RI) profile based on their shape. The antireflection efficiency depends on the refractive index profile, with the quintic RI profile being recognized as ideal for superior antireflection. However, fabricating nano-sized structures with a desired shape, particularly in silicon with a quintic RI profile, has been a challenge. In this study, we introduce a funnel-shaped silicon (Si) ARS with a quintic RI profile. Its antireflective properties are demonstrated through reflectance measurements and by an application to a photodetector surface. Compared to the film Si and cone-shaped ARS types, which are common structures to achieve antireflection, the funnel-shaped ARS showed reflectance of 4.24% at 760 nm, whereas those of the film Si and cone-shaped ARS were 32.8% and 10.6%, respectively. Photodetectors with the funnel-shaped ARS showed responsivity of 0.077 A/W at 950 nm, which is 19.54 times higher than that with the film Si and 2.45 times higher than that with the cone-shaped ARS.

## Introduction

The antireflection effect is a crucial property in optoelectronic applications such as solar cells and photodetectors (PDs), as its effect enhances light absorption by reducing the amount of light that is reflected^1–4^. Light reflection occurs at the boundary where the refractive index (RI) of a medium changes, with the amount of reflection increasing as the degree of the RI change increases^5–7^. Therefore, antireflective structures (ARS) are receiving considerable attention due to their ability to regulate the RI in desirable ways^8–13^. Developing a structural method to regulate this RI change at the interface is now a representative approach to achieving the antireflection effect. ARS can exhibit various RI profiles depending on their shape^14–16^, and it is theoretically known that the RI should adhere to a quintic profile to achieve the ideal antireflection effect^17–19^. To achieve a quintic RI profile in ARS, the design should feature a structure with a narrow upper part that gradually widens towards the bottom, aligning the refractive index to match that of the air at the top and the substrate at the bottom. The transparent nature of the wings of glass-winged butterflies is a notable example of such structures observed in nature^20,21^.

Numerous studies have explored antireflective layers with a quintic RI profile using a variety of materials^22,23^. However, despite the emerging role of silicon (Si) optical transducers in solar cells^9,24,25^ and photodetectors^26–28^, the realization of a quintic RI profile in Si, along with a viable means of fabricating such a profile, has not yet been demonstrated. Particularly for Si, high reflectance arises due to its high RI^29^, which generates a sharp change in the RI at the interface with air. To improve the antireflection effect in Si, previous researchers developed structures consisting of random^30–33^, parabolic^34,35^, vertical rod^36,37^ or pyramid shapes^38–40^ through a diverse range of dry and wet etching methods^41–46^. Despite the extensive research on Si antireflection structures, most studies primarily concentrated on the fabrication of tiny nano-sized structures. However, there has been a lack of consideration regarding which specific shapes of structures should be fabricated for optimal antireflective properties.

In this paper, we introduce Si nanostructures to achieve a quintic RI profile, known to be an ideal profile for the antireflection effect, along with a corresponding fabrication method, demonstrating an improved antireflection effect in the 500–1100 nm wavelength range. Based on the Bruggeman model, we designed funnel-shaped Si nanostructures with a tip-shaped upper section and a triangular lower section. Then, we employed a hybrid process that uses reactive ion etching (RIE) and potassium hydroxide (KOH) etching to fabricate the designed Si nanostructures. We verify the antireflection effect of the proposed structures using Ansys Lumerical finite-difference time-domain (FDTD) simulation, comparing structures of different shapes. Additionally, the reflectance of the proposed funnel-shaped ARS is assessed, and its antireflection effect is demonstrated by comparing it with a film Si and a cone-shaped ARS of the types commonly known as Si ARS^47–49^, showing significant improvements simply when adding a tiny tip to the cone structure. Finally, we applied this structure to PDs and evaluated its performance, demonstrating that the funnel-shaped Si ARS contributes significantly to enhanced performance outcomes.

## Results and discussion

**Figure 1:**
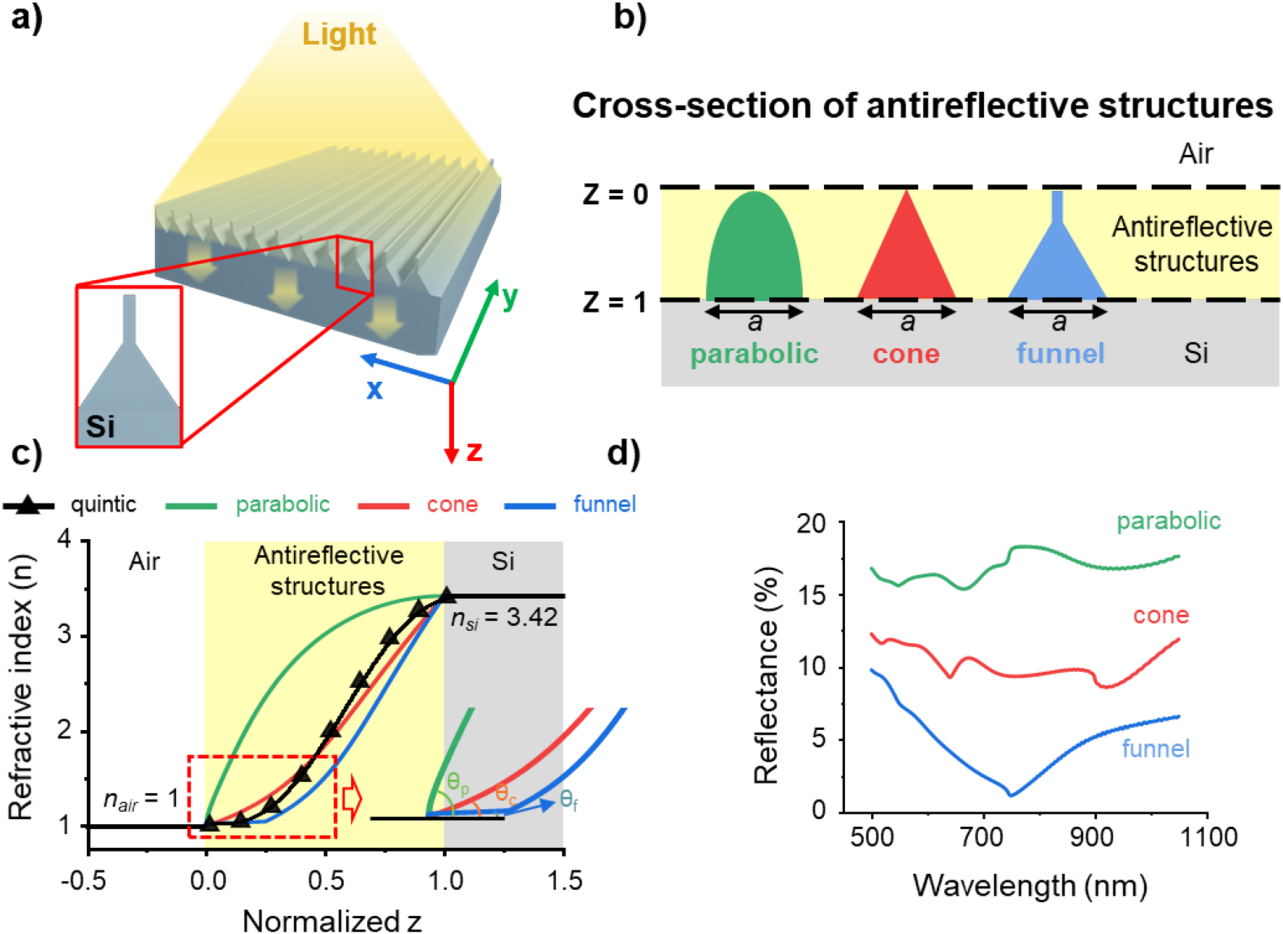
Schematic of the funnel-shaped structures and reflectance based on structural shape. (**a**) The schematic of funnel-shaped silicon (Si) antireflective structure. (**b**) Structures with identical width and height in parabolic, cone, and funnel shapes (a = 400 nm). (**c**) Quintic refractive index profile and refractive index profiles of parabolic, cone, and funnel-shaped structures. (**d**) Simulation results of reflectance for parabolic, cone, and funnel-shaped structures.

The antireflection effect depends on the changes in RI; thus, the design of the ARS shape, which determines its RI profile, is undoubtedly crucial. Therefore, designing the structure's shape is fundamental to achieving a superior antireflection effect. In this study, a Si ARS was designed to achieve a quintic RI profile, known for its minimal rate of RI change at a medium-ARS interface and its gradual, non-abrupt transitions. The structure, designed in a funnel shape, adheres to the quintic RI profile and was constructed in a one-dimensional format to simplify the observation of changes in its cross-sectional shape (Fig. 1a). An analysis of its antireflective performance involved comparing the designed one-dimensional funnel-shaped structure with other one-dimensional structures of the same width (a = 400 nm) and height but in parabolic and cone shapes (Fig. 1b). One-dimensional parabolic, cone, and funnel-shaped structures each possess parabolic, cone, and funnel cross-sectional shapes in the x–z plane, respectively, and extend in the direction of the y-axis. The RI profile for each structure was calculated using the Bruggeman model^50^ (Fig. S1), as shown in Fig. 1c. The RI profile of the antireflection structures varied according to the shape, influenced by the volume ratio of the structure to the medium. Figure 1a–c indicate that the top of the structure, being the initiation point of the RI change from air, plays a vital role in preventing an abrupt RI change for effective antireflection. Therefore, a design with a slimmer and sharper upper part results in a RI that more closely aligns with that of air. It is important to note that in Fig. 1a, as the structure extends downward, it gradually widens to match the RI of Si. The proposed funnel-shaped structure with the quintic RI profile consists of a thin, tip-like upper part and a triangular lower part. The upper part minimizes the RI difference at the air-structure interface, while the lower part, triangular in shape, gradually increases the RI to match that of Si. In Fig. 1c, the black solid line marked with triangle symbols represents the quintic RI profile. The quintic RI profile exhibits superior antireflective properties due to a zero rate of RI change at the air-ARS interface and a quintic curvature from the top to the bottom of the ARS. Similarly, the funnel-shaped ARS also possesses a minimal rate of RI change at the air-ARS interface and exhibits a curvature similar to the quintic profile from top to bottom. The inset in Fig. 1c demonstrates the RI change at the air-ARS interface for parabolic (*θ*_*p*_) cone (*θ*_*c*_), and funnel-shaped structures (*θ*_*f*_), showing that the funnel-shaped structure undergoes significantly smaller RI changes compared to the parabolic and cone-shaped structures at the interface. The reflectance of the structures with different RI profiles was analyzed by means of FDTD simulations (Fig. 1d). The simulations, conducted in a normal incident light, utilized average values for both the transverse magnetic (TM) mode, oscillating along the x-axis, and the transverse electric (TE) mode, oscillating along the y-axis. It was observed that the reflectance of the funnel-shaped structures was lowest compared to both the parabolic and cone-shaped structures within a wavelength range of 500–1100 nm. This lower reflectance of the funnel-shaped structures is attributed to their design, where the upper part exhibits the smallest RI difference with air, closely aligned with the quintic profile.

**Figure 2:**
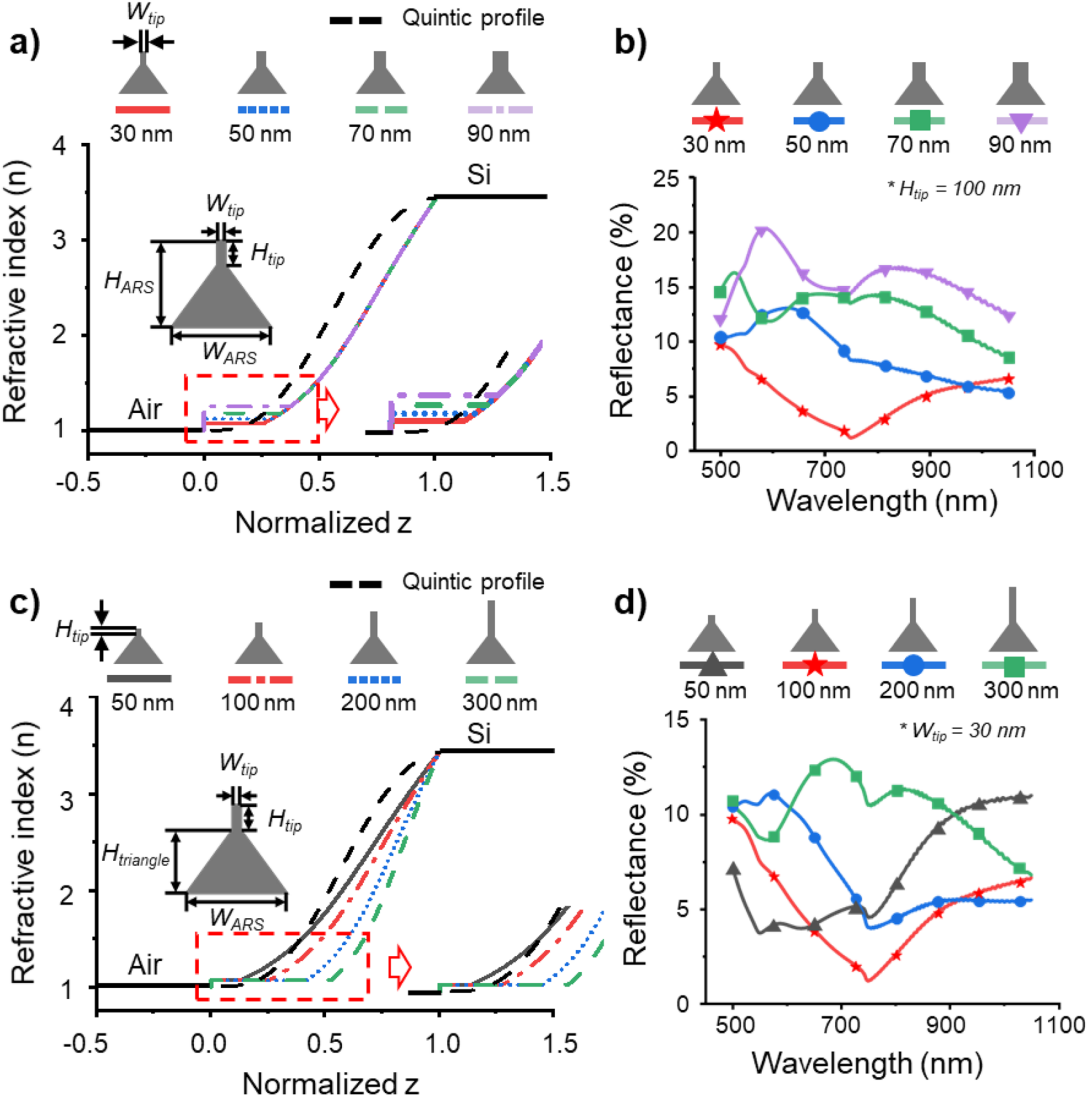
Dimension optimization of the funnel-shaped structures. (**a**) Refractive index profile of the funnel-shaped structures with an upper part width (*W*_*tip*_) ranging from 30 to 90 nm and the quintic refractive index profile. The funnel-shaped structures width (*W*_ARS_) and height (*H*_*ARS*_), and an upper part height (*H*_*tip*_) is fixed at 400 nm, 350 nm, and 100 nm, respectively. (**b**) Simulation results of reflectance for funnel-shaped structures presented in (**a**). (**c**) Refractive index profile of the funnel-shaped structures with an upper part height ranging from 50 to 300 nm and the quintic refractive index profile. The funnel-shaped structures width (*W*_*ARS*_) and lower height (*H*_*triangle*_), and an upper part width (*W*_*tip*_) is fixed at 400 nm, 250 nm, and 30 nm, respectively. (**d**) Simulation results of reflectance for the funnel-shaped structures presented in (**c**).

To optimize the funnel-shape structures to maximize the antireflection effect, we compared the RI profile and reflectance based on the width and height of the upper part of the structures. In the funnel-shaped structures with ARS width (*W*_*ARS*_), height (*H*_*ARS*_), upper height (*H*_*tip*_), and upper width (*W*_*tip*_) of 400 nm, 350 nm, 100 nm, and 30 nm, respectively, the *W*_*tip*_ was increased from 30 to 90 nm. The RI profiles and reflectance were computed using FDTD simulations (Fig. 2a,b). Figure 2a shows an increase in the RI difference at the air-structure interface with an increase in *W*_*tip*_. The FDTD simulation results in Fig. 2b indicate an increase in the reflectance in the wavelength range of 500 nm to 1100 nm as *W*_*tip*_ increases. Structures with a *W*_*tip*_ of 30 nm demonstrated the lowest reflectance due to the smallest RI difference from air. Figure 2c illustrates the RI profiles of the structures as a function of the variance of *H*_*tip*_, indicating that as *H*_*tip*_ increases, the rate of change of the refractive index within the antireflective structure varies. This results in an increased rate of RI change in the lower part of the structure. Figure 2d presents the reflectance of structures as *H*_*tip*_ is varied, calculated from FDTD simulations in the 500–1100 nm wavelength range. These results indicate that structures with an upper width and height of 30 nm and 100 nm, respectively, have the lowest reflectance. This is due to the fact that when *H*_*tip*_ exceeds 100 nm, a shorter *H*_*triangle*_ causes some deviation from the quintic RI profile. Conversely, if *H*_*tip*_ is shorter than 100 nm, the result is a failure to reduce the RI difference sufficiently between the upper part and air. When compared to the quintic RI profile (Fig. S2), the RI profile of the structures with an *H*_*tip*_ value of 100 nm most closely matches the quintic profile (Supplementary Note 1).

**Figure 3:**
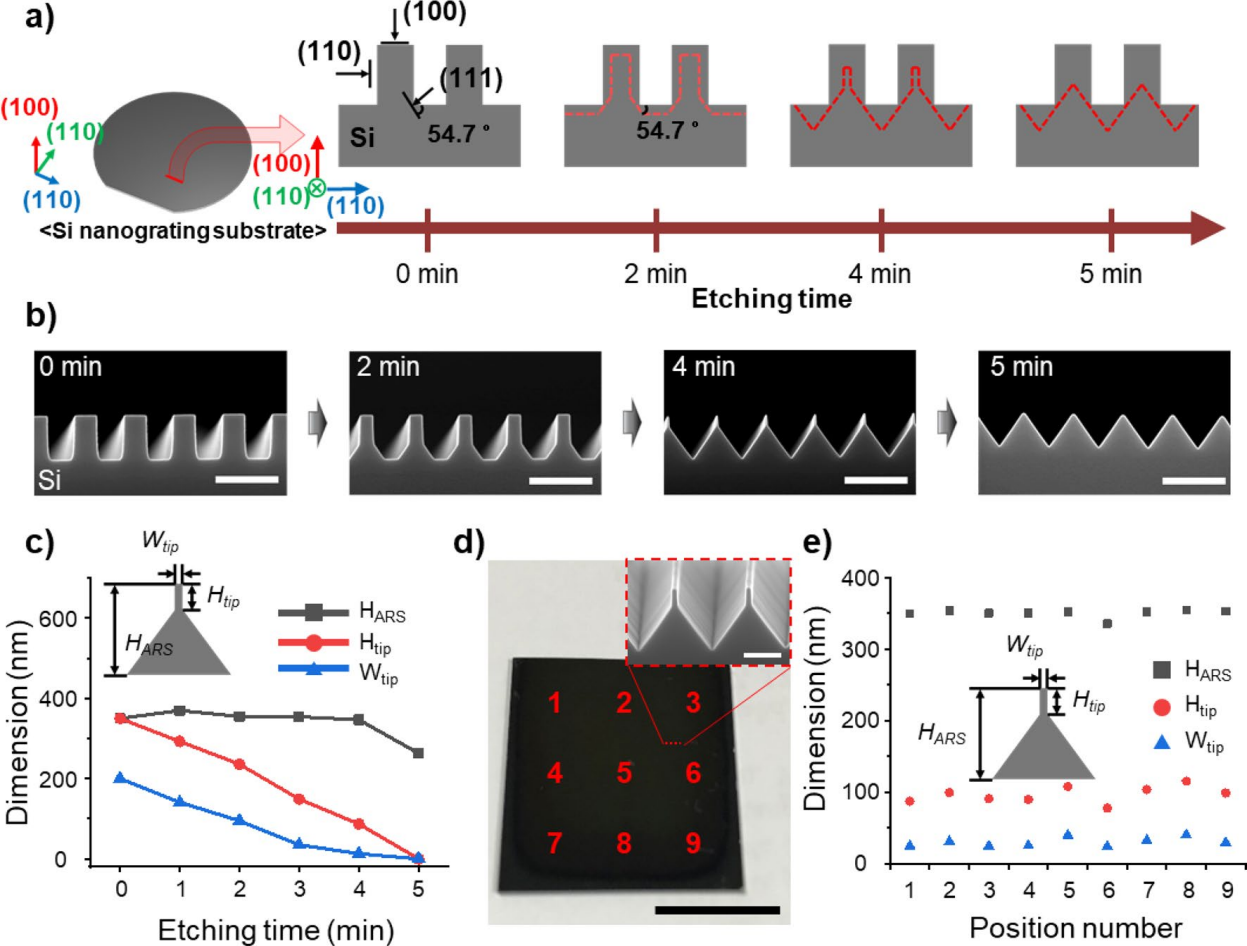
Fabrication process and results of funnel-shaped structures. (**a**) Diagram of the fabrication process for funnel-shaped structures on a Si nanograting substrate. (**b**) Cross-sectional scanning electron microscope (SEM) images of Si structures with different durations of KOH etching (scale bars: 500 nm). (**c**) Structural dimension as a function of KOH etching time. (**d**) Optical microscopy (OM) image of a Si substrate with fabricated funnel-shaped structures (scale bar: 1 cm). Insets are enlarged cross-sectional SEM images of the optimized funnel-shaped structures (scale bars: 200 nm). (**e**) Dimensions of funnel-shaped structures located at positions 1 to 9 on a Si substrate with fabricated funnel-shaped structures.

To fabricate the proposed funnel-shaped structure, a Si nanograting substrate was etched by immersing it in a KOH solution at 65 °C. The Si nanograting substrate was patterned with a 200 nm width and a 400 nm period using KrF lithography, followed by reactive ion etching (RIE) to a depth of 350 nm. The orientation of the Si nanograting was aligned such that its top surface and the direction perpendicular to the nanograting both matched the (110) crystal plane of the silicon substrate. This alignment was crucial as the (111) plane of Si is nearly non-etchable in KOH, whereas the (100) and (110) planes are etchable, allowing for the creation of a funnel shape, as illustrated by the red dashed line in Fig. 3a. For precise control of the funnel shape, the etching rates of Si in various concentrations of KOH solutions were examined (Fig. S3). To etch Si at the nanometer scale per minute, a solution with a 0.45% concentration was prepared by mixing 2 mL of 45% KOH solution with 200 mL of deionized (DI) water, with 150 mL of IPA solution added to reduce the surface roughness. The prepared KOH solution was placed on a hot plate and maintained at a temperature of 65 °C, with the Si nanograting substrate immersed at that point. Over time, the rectangular cross-section of the nanograting transitioned into a shape resembling the red dashed line depicted in Fig. 3a. As shown in the scanning electron microscope (SEM) image in Fig. 3b, the proposed funnel-shaped structures could be etched within four minutes. Extending the etching time beyond this duration resulted in the complete etching of the funnel structure's upper tip, forming a cone-shaped structure. Figure 3c displays the dimensional changes in the structure with the duration of etching in the KOH solution. At up to four minutes of immersion in the KOH solution, the width and height of the upper part decreased linearly, indicating a consistent etching rate and allowing for the fabrication of structures with desired dimensions. A successfully fabricated sample of the optimized antireflection structure with a 30 nm width of the upper part and a 100 nm height of the lower part, as derived from Fig. 2b,d, is shown in Fig. 3d. Cone-shaped structures with a width of 400 nm and height of 330 nm were also fabricated. The uniformity of the funnel-shaped antireflective structures within the fabricated sample was confirmed by measuring the dimensions of the height of the ARS and the width and height of the upper part from positions 1 to 9, confirming considerable uniformity across the sample as the dimensions of the structures at each location were similar to those of the optimized funnel-shaped structure (Fig. 3e).

**Figure 4:**
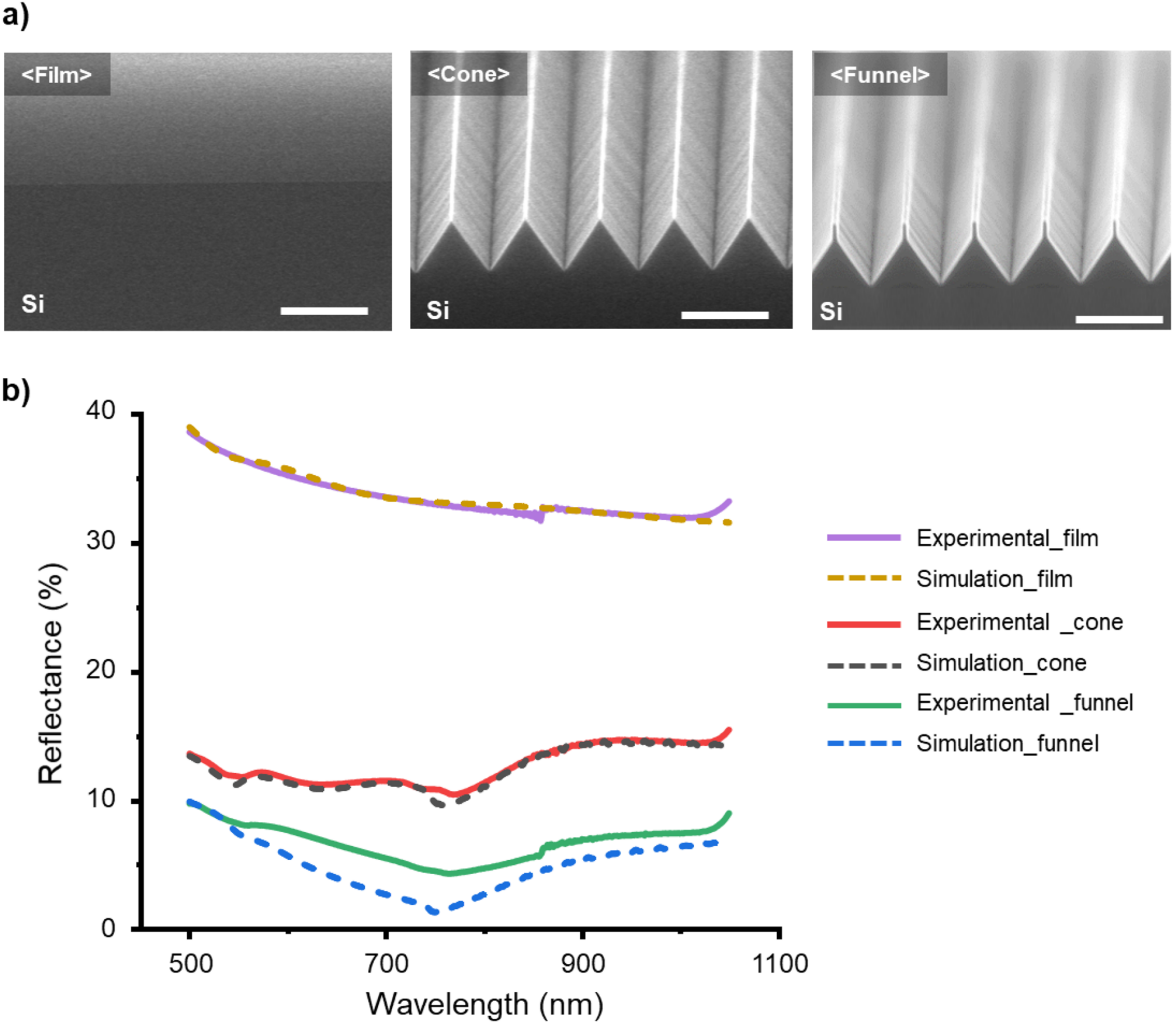
Comparison of reflectance between film, cone-shaped and funnel-shaped structures. (**a**) SEM images at an inclined view of film, cone-shaped and optimized funnel-shaped structures (scale bars: 500 nm), (**b**) Simulation and experimental results of reflectance for fabricated film, cone and funnel-shaped structures.

To demonstrate the antireflection effect of the optimized funnel-shaped structures, a reflectance comparison was conducted, involving film and cone-shaped ARS samples. A reflectance was measured using a Lambda 1050 UV/Vis/NIR spectrometer. Cross-sectional SEM images of the film, cone-shaped ARS and the optimized funnel-shaped ARS are shown in Fig. 4a. These structures, each featuring a 1 nm thick layer of silicon dioxide on their surfaces to account for the presence of native oxide, were simulated for their respective shapes using FDTD simulations (Fig. S4). Their reflectance in the 500–1050 nm wavelength range was measured using a UV–visible-NIR spectrometer. Figure 4b displays the results of the simulations and reflectance measurements of the film, cone-shaped and funnel-shaped ARS specimens, showing a high degree of similarity between the simulation results and the measured reflectance outcomes. The funnel-shaped ARS specimen exhibited significantly lower reflectance compared to the film and cone-shaped ARS types due to the unique upper tip design of the former, which minimizes the refractive index (RI) difference with air at the air-structure interface. At a wavelength of 760 nm, the funnel-shaped ARS exhibits reflectance of 4.24%, while film and cone-shaped ARS types show considerably higher reflectance rates of 32.8% and 10.6%, respectively. This indicates that the reflectance of the funnel-shaped ARS is only 12.9% of that of the film structures and 40% of the cone-shaped ARS, highlighting the significance of reducing the refractive index difference at the air-structure interface for effective antireflection. Moreover, the proposed design displayed less than 10% reflectance across the wavelength range of 500 nm to 1050 nm. The slight discrepancies between the simulated and measured values for the funnel-shaped structures are attributed to minor variations in the dimensions of these structures across the samples.

**Figure 5:**
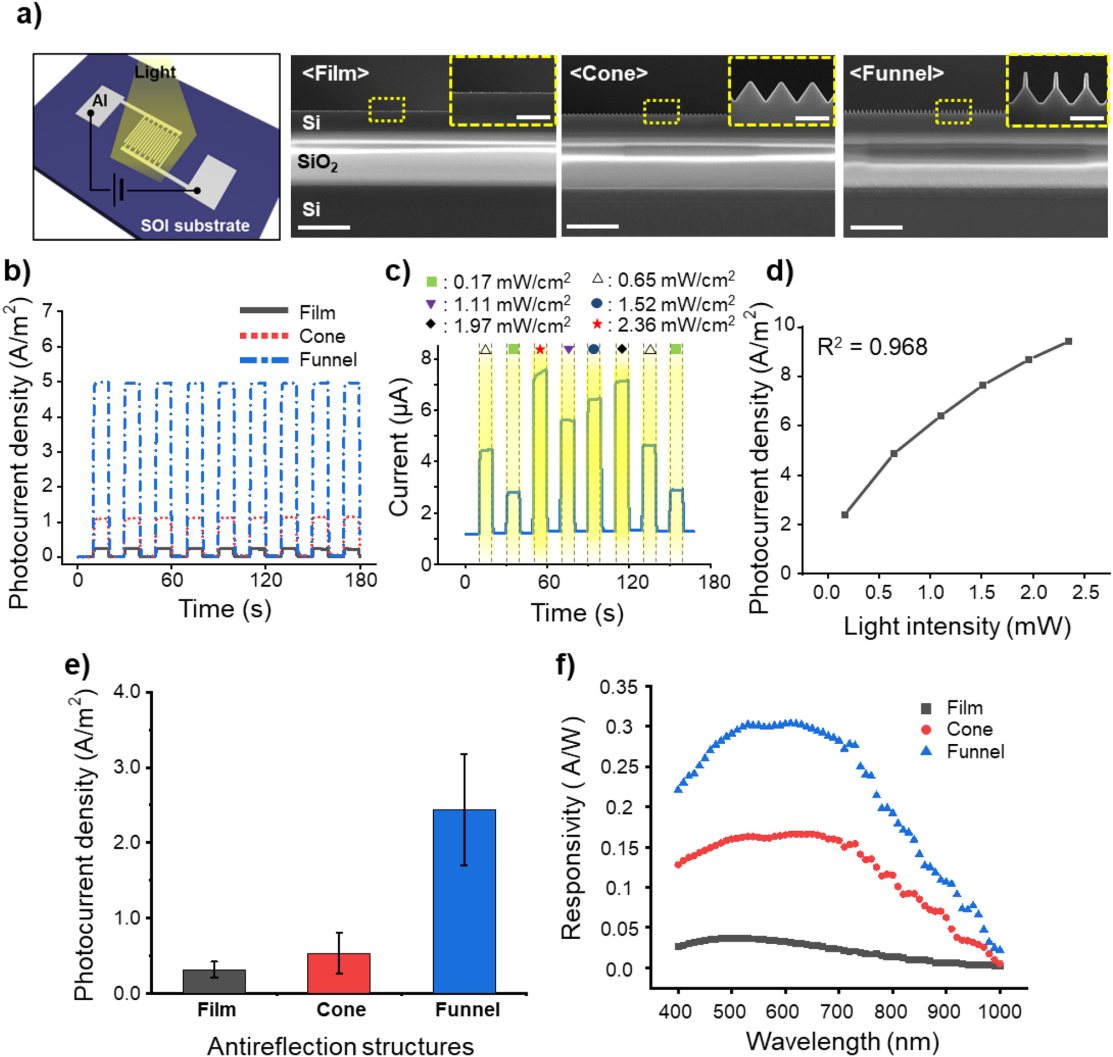
Fabrication results and performance comparison of photodetectors with film, cone-shaped, and funnel-shaped structures. (**a**) Schematic of the photodetector device and cross-sectional SEM images of film, cone-shaped, and funnel-shaped structures fabricated on silicon-on-insulator (SOI) substrate-based photodetectors (scale bars: 5 µm). Insets are enlarged SEM images of the surface structures on the photodetectors (scale bars: 500 nm). (**b**) Photocurrent density of photodetectors with film, cone-shaped, and funnel-shaped structures when exposed to a halogen lamp at 550 nm wavelength with an intensity of 0.65 mW/cm^2^ and under an applied voltage of 0.1 V. (**c**) Current response in the photodetector with funnel-shaped structures when illuminated with a halogen lamp of varying intensities. (**d**) Photocurrent density of the photodetector with funnel-shaped structures in response to varying light intensities. (**e**) Measured photocurrent densities for with film, cone-shaped, and funnel-shaped structures. (**f**) Responsivity of photodetectors with film, cone-shaped, and funnel-shaped structures across the 400–1000 nm wavelength range.

To assess the contribution of the optimized funnel-shaped structures to a performance enhancement in optoelectronics compared to other structures, we evaluated their efficacy by applying the ARS to the surfaces of PDs. PDs were fabricated on silicon-on-insulator (SOI) substrates with a 2-μm-thick Si layer that could maximize the use of photo-generated carriers and minimize the leakage current^51^. On the surfaces of the PDs, structures with film, cone, and funnel shapes were fabricated. The cone-shaped and funnel-shaped structures on the SOI wafer were processed using a method identical to that depicted in Fig. 3a. Interdigitated electrodes made of aluminum were deposited on each structure and photocurrent densities were compared under illumination from a halogen lamp. Figure 5a presents a schematic of the PD and cross-sectional SEM images of each device's surface, confirming the successful formation of both the funnel-shaped structures with a tip width and height of 30 nm and 180 nm, respectively, and the cone-shaped structures. The photocurrent density was measured by alternating the halogen lamp which has an intensity level of 0.65 mW/cm^2^ at a wavelength of 550 nm on and off every ten seconds (Fig. 5b). Upon the application of voltage of 0.1 V, the devices with the film, cone, and funnel-shaped structures exhibited photocurrent densities of 0.242 A/m^2^, 1.1 A/m^2^, and 4.984 A/m^2^, respectively. Notably, the PD featuring the funnel-shaped structures demonstrated a photocurrent density that was 20.59 times higher than that of the film PD and 1.8 times higher than the PD with the cone-shaped structure.

This significant enhancement in the photocurrent density is attributed to the superior antireflection effect of the funnel-shaped structures, which prevents surface reflections and allows more light to be absorbed into the Si, contributing to more photo-generated carriers. The photodetection reliability of the PD with the funnel-shaped structure was also measured while varying the intensity of the halogen lamp over time (Fig. 5c), demonstrating the ability of the proposed design to distinguish different light intensity levels. The linearity of the photocurrent density in response to the intensity of the light was verified for the PD, showing a high coefficient of determination (R^2^) value of 0.968, indicating high detection reliability (Fig. 5d). The coefficient of determination quantifies the predictive accuracy of a statistical model, with its value ranging from 0 to 1. A value approaching 1 indicates increased reliability of the photodetector. Figure 5e displays the photocurrent densities measured for four samples each of the film, cone, and funnel-structure PDs, highlighting the superior performance of the PD with the funnel-shaped structure. The responsivity of each type of PD was measured by applying a voltage of 0.1 V and illuminating with a light source of 50 µW/cm^2^ intensity, across the wavelength range of 400–1000 nm, using the CEP-25ML spectral response measurement system (Fig. 5f), showing how the funnel-structure PDs outperformed the others with a responsivity rate of 0.301 A/W at a wavelength of 550 nm, 9.47 times higher than the film PDs and 1.86 times higher than the cone-structure PDs; the rate was also 0.077 A/W at 950 nm, 19.54 times higher than the film PDs and 2.45 times higher than the cone-structure PDs.

## Conclusion

In conclusion, for the first time, funnel-shaped ARS demonstrating a quintic RI profile in Si, a crucial material in various optical transducers, is successfully designed and fabricated to achieve a high antireflection effect. We fabricated uniform and reliable funnel-shaped structures using RIE and KOH etching methods. The dimensions of these funnel-shaped structures were optimized through FDTD simulations, resulting in structures that exhibited a reflectance rate of 4.24% at a wavelength of 760 nm and less than 10% reflectance in the wavelength range of 500–1050 nm, less than half of the rate of similarly sized cone-shaped ARS. Additionally, when applied to PDs on SOI substrates and exposed to a halogen lamp at a wavelength of 550 nm with an intensity level of 0.65 mW/cm^2^, the PDs with the funnel-shaped ARS demonstrated a photocurrent density of 4.984 A/m^2^, an outcome 20.59 times higher than that of film PDs and 1.8 times higher than that of PDs with cone-shaped structures. Responsivity measurements in the wavelength range of 400–1000 nm revealed that the PDs with the funnel-shaped structures had responsivity of 0.301 A/W at 550 nm, 9.47 times higher than that of the film PDs, and 1.86 times higher than that of the PDs with the cone-shaped structures, also showing an outcome of 0.077 A/W at 950 nm, 19.54 times higher than that of the film PDs and 2.45 times higher than that of the PDs with the cone-shaped structures. We believe that due to the uniform and reliable fabrication methods used here, the application of funnel-shaped structures in Si-based optoelectronic devices, such as solar cells and PDs, can be expected to yield highly enhanced performance outcomes in the near future.

### Supplementary Information

Supplementary Information.

## Data Availability

All data generated or analyzed during this study are included in the manuscript or supplementary information.
