# Supplementary material for: Quintic refractive index profile-based funnel-shaped silicon antireflective structures for enhanced photodetector performance
Source: Sci Rep. 2024 May 6;14:10410. doi: 10.1038/s41598-024-61156-6 (PMC11074249; doi:10.1038/s41598-024-61156-6)
Supplement: Supplementary file 1 — Supplementary Information. [file 41598_2024_61156_MOESM1_ESM.docx]

Supplementary Information

**Quintic Refractive Index Profile-Based Funnel-Shaped Silicon Antireflective Structures for Enhanced Photodetector Performance**

Beom-Jun Kim^1^, Min-Seung Jo^1,2^, Jae-Soon Yang^1^, Myung-Kun Chung^1^, Sung-Ho Kim^1^, and Jun-Bo Yoon^1^*

^1^ School of Electrical Engineering, Korea Advanced Institute of Science and Technology (KAIST), 291 Daehak-ro, Yuseong-gu, Daejeon 34141, Republic of Korea

^2^ Center for Bio-Integrated Electronics, Northwestern University, 633 Clark St, Evanston, IL 60208, United States

E-mail: Jun-Bo Yoon, E-mail: jbyoon@kaist.ac.kr


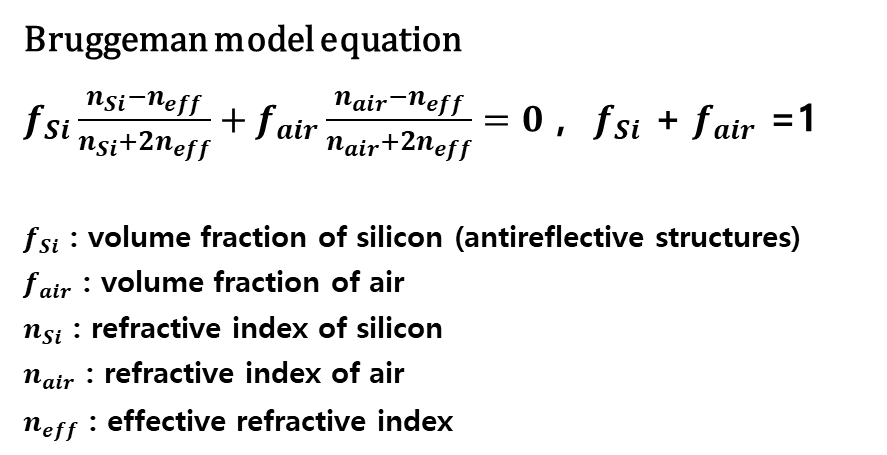


Figure S1. Bruggeman model equation for effective refractive index of antireflective structures.

**[Supplementary Note 1]**

The superior antireflective effect of the quintic profile is attributed not only to the minimal RI difference at the air-structures interface but also to the quintic curvature within the structure. To verify the similarity between the RI profile of the funnel-shaped structures and the quintic curvature, we overlaid the RI profile of a funnel-shaped structure with a tip height of 50 nm, 100 nm, 200 nm, and 300 nm with the quintic RI profile for comparison. Figure S2 reveals that the refractive index profile of funnel-shaped structures with a 50 nm tip height does not closely follow the quintic profile curvature throughout. Additionally, structures with 200 nm and 300 nm tip heights begin to diverge from the quintic profile towards the bottom of the structures. However, funnel-shaped structures with a 100 nm tip height consistently adhere to the quintic profile, showing a significant alignment throughout.


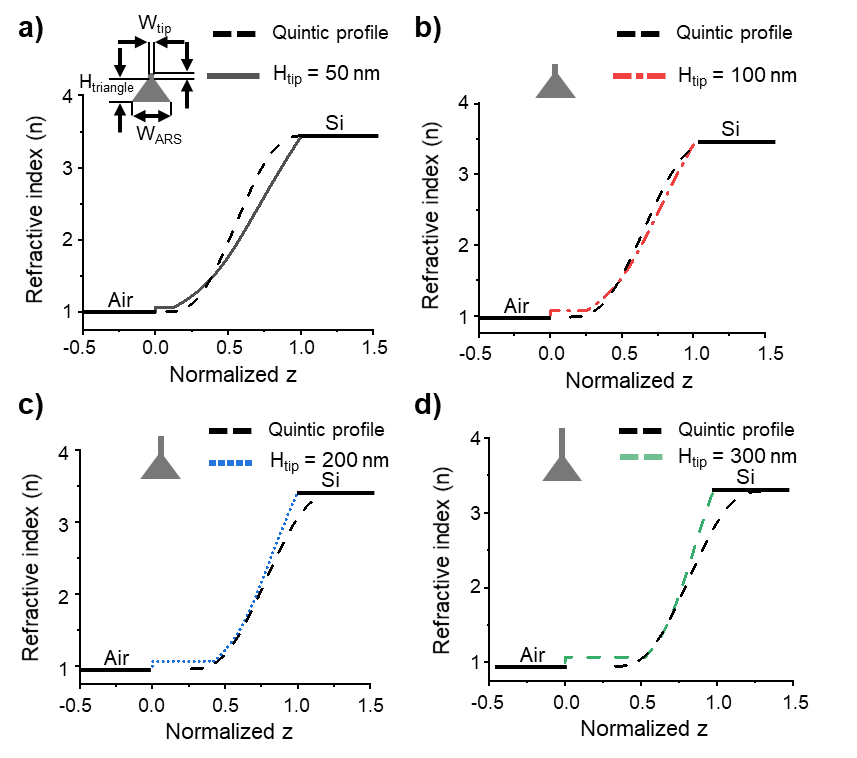


Figure S2. Comparison of the refractive index profile of funnel-shaped structures with tip height of a) 50 nm, b) 100 nm , c) 200 nm, d) 300 nm to the quintic refractive index profile. The funnel-shaped structures width (*W_ARS_*) and lower height (*H_triangle_*), and a tip width (*W_tip_*) is fixed at 400 nm, 250 nm, and 30 nm, respectively.


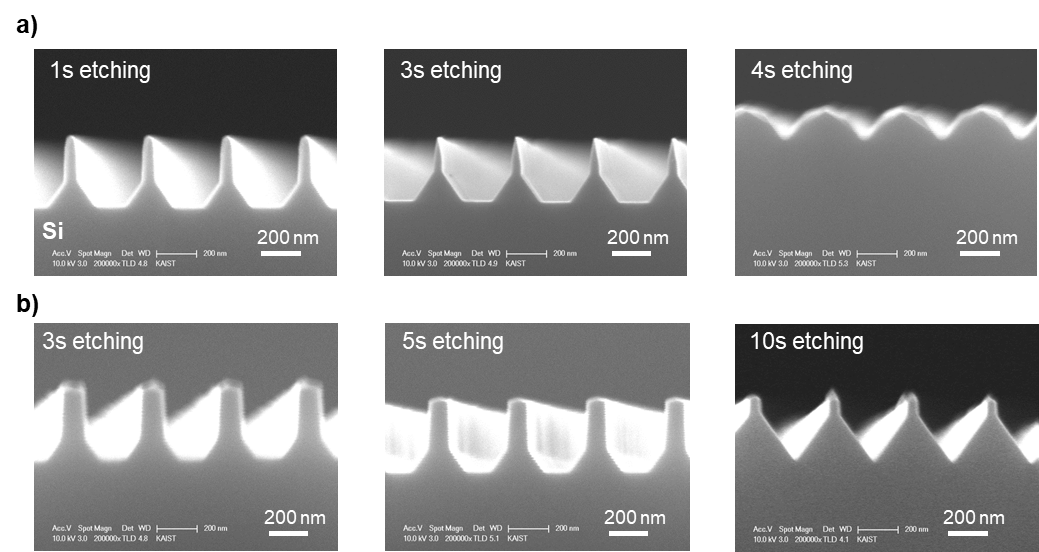


Figure S3. Cross-sectional SEM images of Si nanograting substrates with a width of 200 nm and a period of 400 nm, etched over time in a) a solution of 100 ml of 45% KOH, 200 ml of deionized (DI) water, and 150 ml isopropyl alcohol (IPA), b) a solution of 10 ml of 45% KOH, 200 ml of DI water, and 150 ml of IPA.


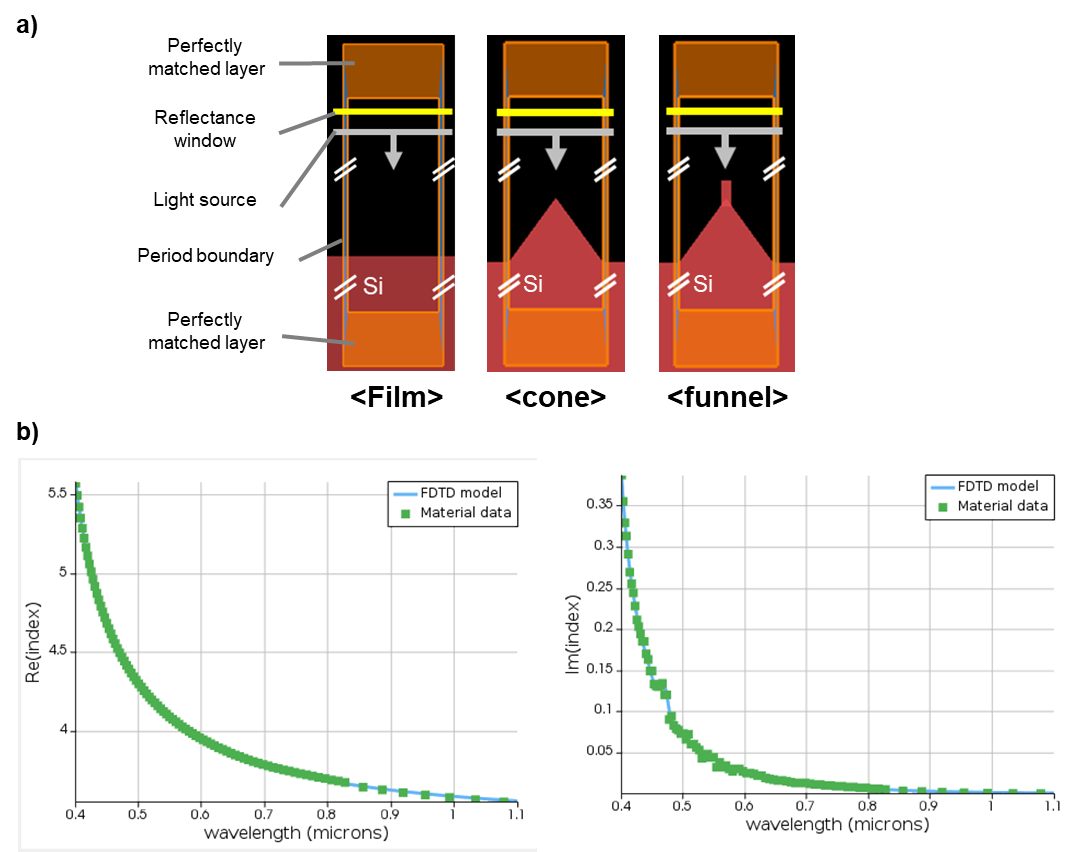


Figure S4. The finite-difference-time-domain (FDTD) simulation settings for a) film, cone-shaped and optimized funnel-shaped structures, b) the refractive index of silicon as a function of wavelength.
